# Performance of PRISM III and PIM 2 scores in a cancer pediatric intensive care unit

**DOI:** 10.5935/0103-507X.20210013

**Published:** 2021

**Authors:** Emmerson Carlos Franco de Farias, Mary Lucy Ferraz Maia Fiuza Mello, Patrícia Barbosa Carvalho Assunção, Alayde Vieira Wanderley, Kissila Márvia Matias Machado Ferraro, Mayara Márvia Matias Machado, Sarah Jennings Marinho

**Affiliations:** 1 Pediatric Intensive Care Unit, Hospital Oncológico Infantil Octávio Lobo - Belém (PA), Brazil.; 2 Pediatric Oncology Unit, Hospital Oncológico Infantil Octávio Lobo - Belém (PA), Brazil.; 3 Pediatric Intensive Care Unit, Fundação Santa Casa de Misericórdia - Belém (PA), Brazil.; 4 Universidade Federal do Pará - Belém (PA), Brazil

**Keywords:** Risk assessment, Prognosis, Child mortality, PRISM, PIM 2, Intensive care units, pediatric, Pediatric oncology, Medição de risco, Prognóstico, Mortalidade da criança, PRISM, PIM 2, Unidades de terapia intensiva pediátrica, Oncologia pediátrica

## Abstract

**Objective:**

To assess the performance of Pediatric Risk of Mortality (PRISM) III and Pediatric Index of Mortality (PIM) 2 scores in the pediatric intensive care unit.

**Methods:**

A retrospective cohort study. Data were retrospectively collected from medical records of all patients admitted to the pediatric intensive care unit of a cancer hospital from January 2017 to June 2018.

**Results:**

The mean PRISM III score was 15, and PIM 2, 24%. From the 338 studied patients, 62 (18.34%) died. The PRISM III estimated mortality was 79.52 patients (23.52%) and for PIM 2 80.19 patients (23.72%), corresponding to a standardized mortality ratio (95% confidence interval: 0.78 for PRISM II and 0.77 for PIM 2). The Hosmer-Lemeshow chi-square test was 11.56, 8df, 0.975 for PRISM II and 0.48, 8df, p = 0.999 for PIM 2. The area under the Receiver Operating Characteristic curve was 0.71 for PRISM III and 0.76 for PIM 2.

**Conclusion:**

Both scores overestimated mortality and have shown a regular ability to discriminate between survivors and non-survivors. Models should be developed to quantify the severity of cancer pediatric patients in Pediatric Intensive Care Units and to predict the mortality risk accounting for their peculiarities.

## Abstract (pt)

**Objetivo:**

Avaliar o desempenho do Pediatric Risk of Mortality (PRISM) III e do Pediatric Index of Mortality (PIM) 2 em unidade de terapia intensiva pediátrica.

**Métodos:**

Estudo de coorte retrospectivo. Os dados retrospectivos foram coletados dos prontuários de todos os pacientes admitidos na unidade de terapia intensiva pediátrica de um hospital infantil oncológico, entre janeiro de 2017 a junho de 2018.

**Resultados:**

A média do PRISM III foi de 15 e do PIM 2 de 24%. Dos 338 pacientes estudados, 62 (18,34%) morreram. A mortalidade estimada pelo PRISM III foi de 79,52 (23,52%) e pelo PIM 2 de 80,19 (23,72%) pacientes, correspondendo a taxa padronizada de mortalidade (intervalo de confiança de 95%) de 0,78 para o PRISM II e 0,77 para o PIM 2. O teste de ajuste de Hosmer-Lemeshow obteve qui-quadrado de 11,56, 8df, com p = 0,975, para PRISM III, e qui-quadrado de 0,48, 8df, p = 0,999, para o PIM 2. Foi obtida área sob a curva Característica de Operação do Receptor de 0,71 para o PRISM III e 0,76 para o PIM 2.

**Conclusão:**

Os dois escores superestimaram a mortalidade e demonstraram poder regular de discriminação entre sobreviventes e não sobreviventes. Devem ser desenvolvidos modelos para quantificar a gravidade de pacientes pediátricos com câncer em unidade de terapia intensiva pediátrica e predizer o risco de mortalidade que contemplem suas peculiaridades.

## INTRODUCTION

Score systems are used to provide benchmarks recognizable by different observers. They are used to indicate the severity and assess the mortality risk in the intensive care unit (ICU). These systems help identify and solve problems and aim to measure the severity of the disease, calibrating that data to a given outcome, such as death or survival. These results are also indicators of the quality of the service provided and useful for internal and external benchmarking.^(1)^

Implementing these systems is highly important for prognostic precision and accuracy in cancer patients admitted to the pediatric intensive care unit (PICU), as this group of patients is characterized by high mortality rates, therefore requiring earlier and effective prediction of untoward outcomes.

Initially, this was a subjective assessment, as in the clinical rating system, where patients were clustered according to their stability and therapeutic intervention requirements.^(2)^

In 1974, Cullen created the Therapeutic Intervention Scoring System (TISS), an indirect and objective method of analyzing the severity of the disease based on therapeutic resources and factors causing clinical worsening of the patient. This method was later reviewed by Keene, in 1983.^(2,3)^ Scores also emerged for specific clinical conditions, such as the Glasgow coma scale and the Injury Severity Score.^(4,5)^

Subjective quantitative scores emerged from the advance of clinical data associated with statistical tools for the determination of relevant clinical variables, allowing mathematical formulas to correlate with percentual mortality risk.^(6)^ Examples of this type of score are the Physiologic Stability Index, which after a revision process originated the Pediatric Risk of Mortality (PRISM).^(7,8)^

The main scores developed for the pediatric population are the PRISM^(9-12)^ and the Pediatric Index of Mortality (PIM)^(13-15)^ and their new versions, PRISM IV^(11)^ and PIM 3.^(13,14)^ These scores were developed by identifying relevant variables for mortality risk and scored after logistic regression statistical analysis.

For Brazil, it estimated 420,000 new cases of cancer during 2019, without considering non-melanoma skin cancer.^(16,17)^ As the median percent of children-youth tumors in the Brazilian Cancer Registry is about 3%, it is assumed that there will be 12,500 new cases of cancer in children and adolescents (up to the age 19).^(18)^

In the recent decades, there has been a marked increase in the overall survival of children with cancer,^(17)^ with five or more years survival rate averaging 58% during the 1970’, and currently above 80% in developed countries.^(19,20)^ However, in developing countries (low and middle-income), the cure expectation remains around 20%.^(21-23)^

These improvements in mortality and survival are accompanied by an increase in complications, such as respiratory and cardiovascular failure, as well as neurological problems, which may require admission to the PICU, where most supportive therapies can be provided.^(24,25)^

The performance of severity scores in children with onco-hematological diseases, besides presenting a wide closely population-related variation in prognosis, also shows scarcity of studies.^(1)^ These divergences raise questions about the use of these scores in pediatric oncology. Unfortunately, even today there is no mortality prediction score specifically developed for pediatric non-bone marrow transplantation cancer patients,^(26)^ despite numerous efforts.

We should point out that during the period of data collection for this study, PRISM IV was not yet in the public domain, and the standard adopted institutionally for this assessment was based on PRISM III and PIM 2.

This study was aimed to assess the performance and internal validation of PRISM III and PIM 2 in a reference hospital in pediatric oncology.

## METHODS

A retrospective cohort study was conducted. The data were retrospectively collected from the medical records of all patients admitted to the PICU of the *Hospital Oncológico Infantil Octávio Lobo* em Belém, Pará, in the Brazilian Amazon region, from January 2017 to June 2018.

Patients admitted to the PICU for longer than 8 hours were included. Patients staying for less than 8 hours or less of 4 hours in case of death; admitted with cardiorespiratory arrest or not achieving vital signs stability in 12 hours; in palliative care or with a do not resuscitate order; or with brain death, were excluded.

The assessed variables constituted three groups: clinical-epidemiological characterization; score system calculation, corresponding to the first 24 hours from admission for analysis of the PRISM score system; and outcome. Demographics and clinical information were included for the sample stratification.

A data bank was assembled using the Excel® 2010 software sheets. The statistical Hosmer-Lemeshow test was used for calibration of the model.^(25)^ The analysis was conducted by dividing the patients into ten mortality risk strata, to compare observed and expected mortality. For discrimination between survivors and deaths, the area under the Receiver Operating Characteristics (ROC) curve was calculated.^(26)^

To quantify the quality of care in the PICU using the mortality score, the standardized mortality ratio (SMR),^(27)^ comparing estimated with observed deaths, was adopted.

This study complied with the Resolution 466/12 of the Brazilian Council of Health and was approved by the Research Ethics Committee of the *Fundação Santa Casa de Misericórdia do Pará* (FSCMPA), opinion number 2.695.187; CAAE 89172218.8.0000.5171.

## RESULTS

During the study period, there were 489 hospitalizations. However, only 338 (69.1%) were included; the 151 (30.8%) excluded cases had incomplete information or did not meet the inclusion criteria. The majority were female (50.9%), median age 8 years, standard deviation ± 5 years, ranging from 3 months to 18 years (Table 1).

**Table 1:** Analysis of sociodemographic, clinical, and therapeutic support variables in patients admitted to the pediatric intensive care unit

Variable	Admission
Gender	338 (100)
Male	166 (49.1)
Age	
1 month to 1 year	25 (7.3)
2 - 5 years	140 (41.3)
6 - 12 years	85 (25.3)
13 - 21 years	88 (26)
Other hospitals	96 (28.5)
Admission diagnosis	338 (100)
Respiratory disorder	77 (22.8)
Cardiovascular disorder	32 (9.5)
Neurological disorder	31 (9.1)
Sepsis/septic shock/multi-organ dysfunction	62 (18.4)
Infection (no sepsis reported)	75 (22.2)
Endocrine-metabolic and nutritional disorder	7 (2.2)
Digestive disorder	17 (5.1)
Hematologic and coagulation disorder	57 (16.8)
Monitoring	53 (15.7)
Renal disorder	15 (4.4)
Length of stay (days)	338 (100)
1	18 (5.3)
2 - 7	178 (52.8)
7 - 14	117 (34.5)
> 14	25 (7.4)
Renal support	10 (100)
Peritoneal	2 (20)
Hemodialysis	8 (80)
Oxygen support at admission	338 (100)
Mechanical ventilation	155 (45.9)
Invasive	310 (91.6)
Non-invasive	13 (8.4)
Spontaneous breathing	
No assistance	18 (9.8)
Face mask	130 (71)
Nasal cannula	35 (19.2)
Vasoactive drug	338 (100)
Yes	104 (30.9)
No	234 (69.1)
Time of mechanical ventilation (days)	189 (100)
0 - 1	42 (22.2)
1 - 3	45 (23.8)
3 - 7	35 (18.5)
> 7	67 (35.4)
Shocks	139 (100)
Septic	56 (40.3)
Hypovolemic	37 (26.6)
Cardiogenic	33 (23.7)
Others	13 (9.3)
*Sepsis continuum*	83 (100)
Sepsis and severe sepsis	27 (32.5)
Septic shock/multi-organ dysfunction	56 (67.5)

Results expressed as n (%).

Most of the patients had a clinical admission (66.7%), presenting with acute leukemia (38%), followed by central nervous system tumors (20%). The most common cancer was acute lymphoblastic leukemia (72.5%), followed by acute myeloid leukemia (24.5%).

The most frequent admission diagnosis was respiratory disorders (22.8%), followed by sepsis/septic shock/multi-organ dysfunction (18.4%), and coagulation disorders and bleeding (16.8%).

Of the 338 studied patients, 62 (18.3%) died, and 38 (61.5%) of these deaths were caused by septic shock and/or multi-organ dysfunction.

Tables 2 and 3 evaluate the similarities in the observed and expected mortality by mortality risk strata, using the Hosmer-Lemeshow goodness-of-fit test for PRISM III - in the first 24 hours, and for PIM 2 estimated from the entire sample of the original score, respectively (chi-square = 11.56; 8df; p = 0.975 for PRISM III; chi-square = 0.48; 8df; p = 0.999 for PIM 2).

**Table 2:** Calibration of the Pediatric Risk of Mortality III scores with the Hosmer-Lemeshow goodness-of-fit test, by mortality and survival risk strata of patients admitted to the pediatric intensive care unit

PRISM III Score	Admission (338) p value = 0.975	Survival		Mortality	
		Observed	Expected	Observed	Expected
< 1	3	3	2.9807	0	0.0193
1 - 5	15	14	14.7598	1	0.2402
5 - 7.5	39	37	37.8253	2	1.1747
7.5 - 10	44	43	41.7476	1	2.2524
10 - 12.5	42	40	38.6120	2	3.3880
12.5 - 15	54	49	49.3062	5	4.6938
15 - 20	60	48	44.3365	12	15.6634
25 - 30	17	4	4.803	13	12.197
> 30	15	6	1.2975	9	13.7025
Total	338	276	258.478	62	79.52197

PRISM - Pediatric Risk of Mortality.

**Table 3:** Calibration of the Pediatric Index of Mortality 2 scores with the Hosmer-Lemeshow goodness-of-fit test, by mortality and survival risk strata of patients admitted to the pediatric intensive care unit

PIM 2 Score	Admission (338) p value = 0,99	Survival		Mortality	
		Observed	Expected	Observed	Expected
< 1	36	35	35.8762	1	0.1238
1 - 5	44	42	42.7990	2	1.201
5 - 10	78	73	72.0547	5	5.9453
10 - 15	39	34	34.0602	5	4.9398
15 - 20	33	30	26.9570	3	6.043
20 - 25	11	10	8.4690	1	2.531
25 - 30	10	9	7.1650	1	2.835
30 - 40	10	6	6.5020	4	3.498
40 - 50	9	5	4.7210	4	4.279
> 50	68	32	18.0470	36	49.95301
Total	338	276	257.8098	62	80.19025

PIM - Pediatric Index of Mortality.

Mean scores were PRISM III 15% and PIM 2, 24%. Median PIM 2 and PRISM III for survivors and non-survivors were 2.3 (0.6 - 7.8%) and 13.4% (6.5 - 62%) and 2.8 (1.4 - 9.1%) and 18.7% (6.2 - 55.9%), respectively. However, no statistically significant difference was identified between the groups (p > 0.05) with the Mann-Whitney’s U test.

**Figure 1:**
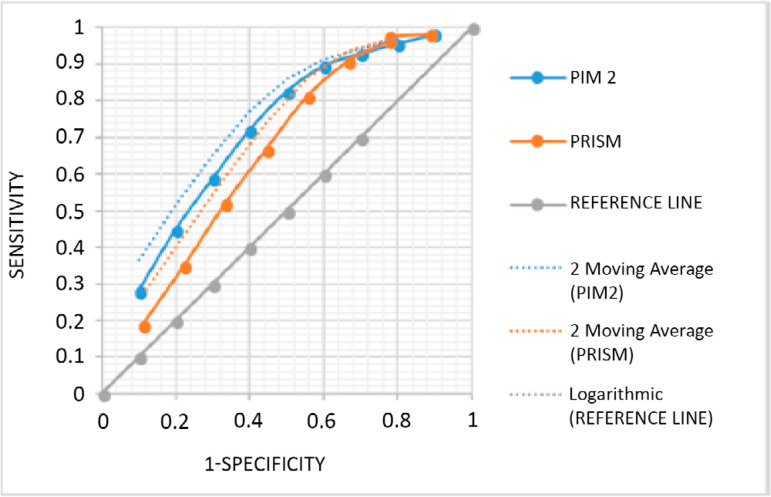
Receiver Operating Characteristic curve overlap: 0.71 (95% confidence interval: 0.47 - 0.92) for Pediatric Risk of Mortality III and 0.76 (95% confidence interval: 0.58 - 0.89) for Pediatric Index of Mortality 2 of patients admitted to the pediatric intensive care unit.

The area under the ROC curve (AUC) was 0.71 (95% confidence interval - 95%CI: 0.47 - 0.92) for PRISM III and 0.76 (95%CI: 0.58 - 0.89) for PIM 2 (Figure 1).

PRISM - Pediatric Risk of Mortality; PIM - Pediatric Index of Mortality.

The PRISM III estimated mortality was 79.5 (23.5%) and PIM 2 80.1 (23.7%) patients. This corresponds to an SMR of 0.78 (95%CI: 0.70 - 0.87) for PRISM III and 0.77 (95%CI: 0.69 - 0.88) for PIM 2.

## DISCUSSION

Regarding the performance of the score concerning the overall population mortality through the SMR, both (PRISM III and PIM 2) overestimated it. Both scores were created some years ago and may have not considered the current population of children and adolescents with complex chronic illness, which may have influenced this difference between the observed and expected mortality. Some studies have found similar results.^(27)^

Evaluation of the discriminatory performance of the models using the area under the ROC curve evidenced that both PRISM III and PIM 2 have a regular ability to discriminate between survivors and non-survivors (0.71 for PRISM III and 0.76 for PIM 2). Many authors have reported that PRISM III overestimates^(27)^ mortality and fails to have good calibration and discrimination in specific populations.^(28-30)^

The study population had an overall mortality rate of 18.3% and, in this percentage, 61.5% were due to septic shock/multi-organ dysfunction. Other studies have shown mortality rates close to this or higher.^(31)^ The development of potentially serious infections is probably associated with the degree of immunosuppression, resulting from both the underlying neoplastic disease and the post-chemotherapy condition.^(32,33)^ It is also important to emphasize that during the study period, sepsis protocols and care-related infection preventive bundles had not yet been implemented. This may have contributed to this higher mortality rate.

This study has limitations. Because it was based on retrospective medical records review, a bias in collection and interpretation must be considered; and also, because it is a single-center study. Additionally, a large portion of patients (30.8%) were excluded from the study. However, as strengths, the study had a moderate sample size and is a pioneer in the region.

The literature still lacks studies evaluating the outcome of pediatric cancer patients admitted to the PICU. In cancer patient care, it is necessary to develop models to quantify the severity of the disease and to predict the mortality risk, accounting for their peculiarities. In the future, the use of these models may be useful to provide better predictions of the disease’s course.

## CONCLUSION

In the oncology pediatric intensive care unit, both scores overestimated the actual mortality over the predicted one. The predictive models studied have shown a regular ability to discriminate between survivors and non-survivors among patients with children and youth cancer. PIM 2 was superior to PRISM III. Therefore, these are important tools for the prognostic assessment of these patients. It is important to emphasize that this was the first study of its kind to be carried out in this specific population sample, and additional research is required for better calibration and validation of these scores in this population.
